# Periodontal Pathogens Promote Oral Squamous Cell Carcinoma by Regulating ATR and NLRP3 Inflammasome

**DOI:** 10.3389/fonc.2021.722797

**Published:** 2021-09-30

**Authors:** Yufei Yao, Xin Shen, Maolin Zhou, Boyu Tang

**Affiliations:** ^1^ State Key Laboratory of Oral Diseases, National Clinical Research Center for Oral Diseases, West China Hospital of Stomatology, Sichuan University, Chengdu, China; ^2^ Department of Cariology and Endodontics, West China Hospital of Stomatology, Sichuan University, Chengdu, China

**Keywords:** OSCC, NLRP3 inflammasome, ATR-Chk1, *P. gingivalis*, *F. nucleatum*

## Abstract

Periodontitis is closely related to oral cancer, but the molecular mechanism of periodontal pathogens involved in the occurrence and development of oral cancer is still inconclusive. Here, we demonstrate that, *in vitro*, the cell proliferation ability and S phase cells of the periodontitis group (colonized by *Porphyromonas gingivalis* and *Fusobacterium nucleatum*, P+) significantly increased, but the G1 cells were obviously reduced. The animal models with an *in situ* oral squamous cell carcinoma (OSCC) and periodontitis-associated bacteria treatment were constructed, and micro-CT showed that the alveolar bone resorption of mice in the P+ group (75.3 ± 4.0 μm) increased by about 53% compared with that in the control group (48.8 ± 1.3 μm). The tumor mass and tumor growth rate in the P+ group were all higher than those in the blank control group. Hematoxylin–eosin (H&E) staining of isolated tumor tissues showed that large-scale flaky necrosis was found in the tumor tissue of the P+ group, with lots of damaged vascular profile and cell debris. Immunohistochemistry (IHC) of isolated tumor tissues showed that the expression of Ki67 and the positive rate of cyclin D1 were significantly higher in tumor tissues of the P+ group. The qRT-PCR results of the expression of inflammatory cytokines in oral cancer showed that periodontitis-associated bacteria significantly upregulated interleukin (IL)-6, tumor necrosis factor (TNF)-α, IL-18, apoptosis-associated speck-like protein containing a CARD (ASC) (up to six times), and caspase-1 (up to four times), but it downregulated nuclear factor (NF)-κB, NOD-, LRR- and pyrin domain-containing protein 3 (NLRP3), and IL-1β (less than 0.5 times). In addition, the volume of spleen tissue and the number of CD4+ T cells, CD8+ T cells, and CD206+ macrophages in the P+ group increased significantly. IHC and Western blotting in tumor tissues showed that expression levels of γ-H2AX, p-ATR, RPA32, CHK1, and RAD51 were upregulated, and the phosphorylation level of CHK1 (p-chk1) was downregulated. Together, we identify that the periodontitis-related bacteria could promote tumor growth and proliferation, initiate the overexpressed NLRP3, and activate upstream signal molecules of ATR-CHK1. It is expected to develop a new molecular mechanism between periodontitis-related bacteria and OSCC.

## Introduction

Oral cancer is the sixth most prevalent malignancy in the world, and oral squamous cell carcinoma (OSCC) accounts for approximately 90% of all oral malignancies, leading to significant morbidity and mortality (1). Carcinogenesis of OSCC is an intricate multistep process initiated by varied pathogenic factors, including genetic predisposition, bad habits, viral infections, and so on. While the possible molecular mechanisms in OSCC are being determined, the definitive reason for the initiation and development of OSCC is still unclear (2).

With the establishment and development of the field of “metagenomics,” more and more in-depth research results suggest that the occurrence and development of OSCC were closely related to the oral symbiotic microbiome that occupied 90% of the human cell composition and exceeded the genetic information of the human genome 100 times. Periodontitis, a dental plaque-induced inflammatory disease, led to irreversible loss of attached tissues and alveolar bone and increases the risk of developing inflammatory tumors (3–6). Evidence has accrued that there might be a close relationship between suspected periodontal pathogen infection, periodontitis, and OSCC. The analysis of large clinical samples showed that the risk of oral cancer and gastrointestinal cancer was significantly increased in periodontal patients (5, 6). Clinically, the presentation of periodontitis was similar to some oral cancers, such as gingival cancer. In some cases, periodontal disease and gingival cancer occurred simultaneously at the same site. Moreover, a meta-analysis showed that the severity of periodontitis was significantly correlated with precancerous lesions and oral cancer (6). The results of metagenomics also showed that there are significant differences in bacterial composition between OSCC and non-tumor tissues. The difference of microbial communities between superficial and deep cancer tissue sites was mainly due to the different glycometabolism and acid tolerance to adapt to the different microenvironments (3, 7). The research now believed that the poor microenvironment of periodontitis was mainly triggered by *Porphyromonas gingivalis*. In the process of reshaping a disease-causing microbiome, *P. gingivalis* was believed to play a key role in the breakdown of homeostasis (4, 8). In addition, *Fusobacterium nucleatum*, one of the periodontal pathogens, promoted the proliferation ability of Tca8113 by causing DNA damage *via* the Ku70/p53 pathway, thereby promoting OSCC (9).

However, the functional manifestation of its core microbial community and its influence mechanism on the occurrence and development of tumors were still unclear. As we all know, inflammation and persistent infection might contribute to various human malignancies and the inflammasome-mediated immune mechanisms, and the monitoring mechanisms based on DNA damage responses (DDRs) played an important role in the progression of inflammatory tumors (10), failure of which led to accumulation of DNA damage and genomic instability. And mounting evidence has suggested that bacterial infections can elicit DNA damage in host cells. In order to ensure the integrity of the genome after cell injury, the cell usually activated a checkpoint mechanism to prevent the progression of the cell cycle. This response depended on two main phosphatidylinositol-3 kinase (PI3K)-related kinases, namely, ataxia telangiectasia-mutated (ATM) and ATM and Rad-3-related (ATR). The ATM–CHK2 pathway played an important role in the G1, S, and G2 phases and is extremely fast and sensitive to DNA double-strand breaks (DSBs), recruiting chromatin binding proteins such as H2AX, MDC1, BRCA1, RNF8, and 53BP1 to the double-strand break point rapidly. While the ATR–CHK1 pathway specifically acted on the S/G2 phase and mainly mediated the replication stress. TopBP1 and Rad9-Hus1-Rad1 were the key protein factors for the activation of the ATR–CHK1 pathway. TopBP1 could activate ATR kinase through binding to the ATR activation domain, triggering phosphorylation of downstream molecules including CHK1. H2AX is a variant of the H2A protein family, which can be phosphorylated by kinases such as ATM and ATR in the PI3K pathway, forming γ-H2AX that can be used as a biomarker for DDR (6, 11).

Inflammasomes are a group of cytosolic protein complexes that are formed to mediate host immune responses to microbial infection and cellular damage. Assembly of an inflammasome triggers proteolytic cleavage of dormant procaspase-1 into active caspase-1, which converts the cytokine precursors pro-interleukin (IL)-1β and pro-IL-18 into mature and biologically active IL-1β and IL-18, and also induces pyroptosis, respectively (12). The NOD-, LRR- and pyrin domain-containing protein 3 (NLRP3) inflammasome has been under intensive investigation given its possible involvement in several human diseases, including immune inflammatory diseases and tumors. Now, data have suggested that there is a close relationship between NLRP3 inflammasome polymorphisms and different malignancies such as colon cancer and melanoma (13). In addition, during the infection process, microorganisms can utilize or hijack the biological functions of the infected cells, causing the accumulation of host cell genome mutations and DNA breakage, which can eventually lead to malignant transformation of cells and tumor formation (14).

Our previous results of Illumina sequencing on the subgingival plaque samples from patients with periodontitis and healthy population found that *Fusobacterium* and *Porphyromonas* were significantly increased in the periodontitis cohort (15). Therefore, the representative strains of *F. nucleatum* and *P. gingivalis* were selected to carry out an in-depth study on the effect and regulatory mechanism of NLRP3 and DDR of oral epithelial cells under the condition of imbalance of oral ecology. We had successfully constructed the model of periodontitis and identified that the periodontitis-related bacteria (*P. gingivalis* and *F. nucleatum*) could promote oral tumor growth and proliferation by initiating the overexpressed NLRP3 and activating upstream signal molecules of ATR-CHK1, which was innovative in the analytical elucidation of the possible linkages and regulatory mechanisms of periodontal bacterial infection–periodontitis–OSCC.

## Materials and Methods

### Bacteria and Culture Conditions

*P. gingivalis* ATCC 33277 and *F. nucleatum* ATCC 25586 were grown in brain heart infusion (BHI; BD, Basingstoke, USA) blood agar plate supplemented with hemin (5 μg ml^−1^) and menadione (1 μg ml^−1^) and 5% defibrinated sheep blood under anaerobic conditions at 37°C overnight. Bacteria were harvested in the late exponential growth phase by centrifugation for 10 min at 4,000×g and 4°C, washed three times with sterile phosphate buffered saline (PBS) before use. According to the relative abundance of previous results of Illumina sequencing, the mixed ratio of the *P. gingivalis* and *F. nucleatum* in periodontitis group (P+) was 1:1. A mixture of 10^9^ colony-forming unit (CFU) of bacteria was used in animal experiments.

### Culture of Cells

HSC-3 (human oral squamous carcinoma cell line) and SCC-7 (murine squamous cell carcinoma cell line) were kindly provided by Prof. Qianming Chen from State Key Laboratory of Oral Diseases, West China Hospital of Stomatology, Sichuan University. Cells were grown in Dulbecco’s modified Eagle’s medium (DMEM; HyClone, Logan, UT, USA) supplemented with 10% fetal bovine serum (FBS; Gibco, Grand Island, NY, USA) at 37°C in a humidified incubator with 5% CO_2_. HSC-3 cells were used at ~75% confluence. Here, 1 × 10^8^/ml SCC-7 cells (5 × 10^6^ SCC-7 cells in 50 μl DMEM) were constructed for transplanting tumors in mice.

### Cell Treatment

The mixture of *P. gingivalis* and *F. nucleatum* was resuspended in an appropriated volume of cell culture medium to achieve the desired bacteria–cell ratio at a multiplicity of infection (MOI) of 200 for the indicated time. Although MOI 100 for infection was commonly used, we increased to MOI 200 due to short-term effects of bacteria (16). For Western blot assay, after infection for 8 h, the cells were washed three times and incubated in fresh DMEM supplemented with metronidazole (200 μg/ml) and gentamicin (300 μg/ml) for 12 h. Then, cells were incubated in fresh DMEM until 24 and 48 h post-infection.

### Cell Proliferation Assay

The effects of bacteria on the viability and proliferation of HSC-3 cells were determined using a Cell Counting Kit-8 (CCK-8; Dojindo, Kumamoto, Japan). Briefly, HSC-3 cells were plated in 96-well plates at 2 × 10^3^ cells per well in the growth medium. Then, 10 μl of CCK-8 (5 mg/ml) was added to each well after bacterial infection at 4, 6, 12, and 24 h. The cells were incubated for 1 h, and OD_450_ was measured using a Varioskan Flash microplate reader (Thermo Fisher Scientific). Finally, the OD_450_ values were converted to cell viability by utilizing standard curves. Each experiment was performed in quadruplicate and repeated at least twice.

### Cell Cycle Analysis

The mixture of *P. gingivalis* and *F. nucleatum* was resuspended in an appropriated volume of DMEM to achieve the desired HSC-3 bacteria–cell ratio at a MOI of 200 for 8 h. Infected or control HSC-3 cells were trypsinized, washed with cold PBS, and then fixed in 70% ethanol at 4°C overnight. The cells were then incubated with RNase A at 37°C for 30 min and stained with cell cycle detection kit (KeyGEN, Jiangsu, China). Cell cycle was assayed using a flow cytometer (Beckman Coulter, Brea, CA, USA).

### Apoptosis Analysis

For annexin V staining, cells were harvested and stained with an annexin V–fluorescein isothiocyanate (FITC) apoptosis detection kit (KeyGEN, Jiangsu, China) according to the manufacturer’s protocol, and flow cytometric analysis (Beckman Coulter) was performed.

### Animal Model

Eight-week-old Balb/c male mice were obtained from where they were group-housed (four mice per cage) in a specific pathogen-free controlled environment. After 1 week, all mice were randomized into two groups: one group was colonized by *P. gingivalis* and *F. nucleatum* (P+) and another served as control. Mice were treated with antibiotic (1 g/L ampicillin, 1 g/L metronidazole, 1 g/L neomycin, 0.5 g/L vancomycin) by the oral route in a volume of 25 μl for 3 days (17). Antibiotic treatment was stopped for 1 day. For P+ group, 200 μl of a mix of bacteria in 2% carboxymethyl cellulose (CMC) was applied at the surface of the mandibular molar teeth, four times a week, for 1 month. The control group was treated with vehicle (CMC) alone. Then, 5 × 10^6^ SCC-7 cells in 50 μl DEME without FBS were injected into the submucosa of the right cheek after 3 weeks of bacterial colonization. After the tumor inoculation, the longest and shortest diameter of the mass was measured in three directions, and primary tumor growth or formation was evaluated for 3 weeks. All mice were sacrificed on 15 weeks, and their tumors were harvested and weighed. These tumor tissues were processed for Western blot, histological examination, immunostaining, and real-time PCR (including spleen). Mandibular bone was fixed in 10% formalin for micro-CT test. Animal experiments were approved by the ethics committee of West China Hospital of Stomatology, Sichuan University (WCHSIRB-D-2020-394).

### MicroCT Analysis

Mandible was dissected after euthanasia and scanned with a micro–computed tomography (μCT50, SCANCO) at the voxel resolution of 10 μm. The amount of alveolar bone was evaluated as previously described by using ImageJ. Mesial and distal bone losses were quantified by measuring the distance between the alveolar bone crest and cement–enamel junction with sagittal images selected in the middle of the tooth. A minimum of four mandibular samples from different mice were examined per group with the analysis performed three times.

### Western Blotting

The proteins of tumor tissues were isolated from snap-frozen tissue samples. Samples for Western blotting were tested by the Total Protein Extraction Kit (SAB, USA) following the manufacturer’s instructions. One volume of 4× sodium dodecyl sulfate (SDS) sample buffer was added, and samples were boiled prior to separation of proteins on 10% SDS–polyacrylamide gel electrophoresis (PAGE). After transfer of proteins, the polyvinylidene fluoride (PVDF) membranes were blocked in 5% non-fat dry milk in Tris-buffered saline (TBS) containing 0.05% Tween-20. The antibodies were bought from Cell Signaling Technology and used at the following dilution: γ-H2AX (1:1,000), ATR (1:1,000), p-ATR (1:1,000), CHK1 (1:1,000), p-CHK1 (1:1,000), RAD51 (1:1,000). Replication protein 32 (RPA32; 1:500) came from Santa Cruz Biotechnology, and the internal control, α-tubulin (1:6,000), came from Proteintech.

### Tumor RNA Isolation and Quantitative Real-Time PCR

Analysis of gene expression was performed by qRT-PCR. Total RNA was isolated from snap-frozen tissues using TRIzol (Invitrogen), according to the manufacturer’s instructions, quantified and evaluated by spectrophotometry. Reverse transcription of RNA into cDNA was performed with the PrimeScript RT Reagent Kit with gDNA Eraser (RR047A; Takara Bio). Real-time polymerase chain reaction was performed with the StepOnePlus Real-time PCR System (Applied Biosystems). The expression levels of IL-6, tumor necrosis factor (TNF)-α, nuclear factor (NF)-κB, NLRP3, apoptosis-associated speck-like protein containing a CARD (ASC), caspase-1, IL-1β, and IL-18 were measured with the glyceraldehyde 3-phosphate dehydrogenase (GAPDH) gene as internal control. All primers in the assay were designed by Primer-BLAST online (National Center for Biotechnology Information, https://www.ncbi.nlm.nih.gov/tools/primer-blast/), and its PCR amplification efficiency reached 90%–110%. The PCRs contained 1 μmol/L for each primer pair and 1 μl cDNA sample in a 25-μl volume. The PCR program is composed of a 5-s preincubation at 95°C. Amplification was achieved with 39 cycles of 5-s denaturation at 95°C, 30-s annealing at 60°C, and 5-s extension at 72°C. All experiments were performed in triplicate. Relative expression was calculated using a 2^–ΔΔCT^ method by normalizing with GAPDH as the internal control. The forward/reverse primer sequences were listed in **Supplementary Table S1**.

### Histochemistry, Immunohistochemistry, and Immunofluorescence

From tumor and spleen tissues after paraffin-embedding, serial sections of 4-μm thick were made and stained with hematoxylin–eosin (H&E) and immunohistochemistry (IHC). The following antibodies were used: Ki67, cyclin D1, γ-H2AX, CD4, CD8 (all from Cell Signaling Technology), and CD206 (Alexa Fluor 488, Servicebio, China). After the slides were deparaffinized and rehydrated, antibodies were applied according to the manufacturer’s protocol.

### Statistical Analysis

SPSS software (version 19.0; IBM Corporation, Armonk, NY, USA) and GraphPad Prism software (version 6; GraphPad Software, San Diego, CA, USA) were used for statistical analysis. Normal data distributions were analyzed by the Kolmogorov–Smirnov test. If the data are not normally distributed, data were analyzed by nonparametric tests. The normally distributed data were presented as the mean ± SD, and one-way analysis of variance was used to compare the means. Student’s t-test was used for pairwise comparisons between groups. Chi-square test was used to analyze clinical samples, and p < 0.05 was considered statistically significant.

## Results

### Effect of Coinfection of Periodontitis-Associated Bacteria on Biological Behavior of HSC-3 Cells

**Figure 1:**
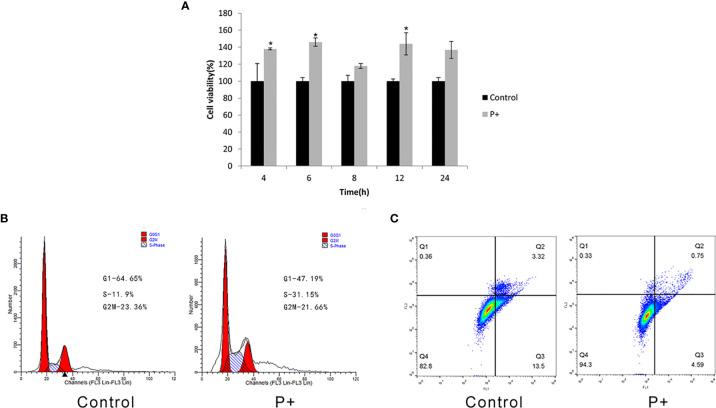
Effect of coinfection of periodontitis-associated bacteria on biological behavior of HSC-3 cells. **(A)** The cell viability assays of HSC-3. *p < 0.05. **(B)** The cell cycle analysis and the specific percentage of G1, S, and G2/M phase cell populations. **(C)** Apoptosis detection.

As shown in **Figure 1A**, compared with the control group, the cell proliferation ability of the P+ group significantly increased from 4 h and was about 1.4 times that of the control group between 6 and 12 h (p < 0.05). In addition, the cell proliferation of P+ group decreased slightly at 24 h. Periodontitis-associated bacteria stimulated HSC-3 cells with MOI = 200 for 8 h. Then, the cells were collected to test the cell cycle and evaluate the percentage of G1, S, and G2/M phase cell populations. As shown in **Figure 1B**, compared with the control group, a significant accumulation of the percentage of S phase cells in the P+ group was observed (from 11.9% to 31.15%). However, the G1 cells were obviously reduced (from 64.65% to 47.19%). Moreover, the apoptosis rate of P+ was 0.75%, which was significantly lower than 3.32% of the rate of control cells (**Figure 1C**).

### Effect of Periodontitis-Associated Bacteria on Alveolar Bone Resorption and Tumor Histology in Tumor-Bearing Mice

**Figure 2:**
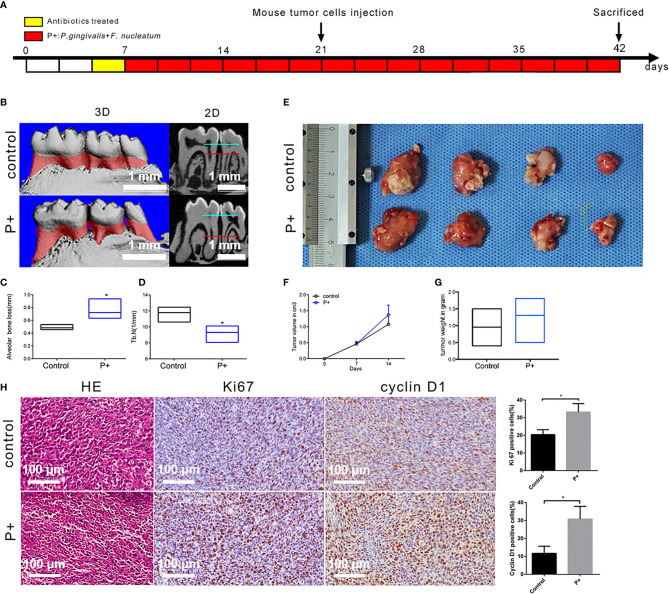
Effect of periodontitis-associated bacteria on alveolar bone resorption and tumor histology in tumor-bearing mice. **(A)** Flowchart of the experimental design. **(B)** The microCT analysis. **(C)** Measurement of the alveolar bone resorption of mice. *p < 0.05. **(D)** Measurement of the number of trabecular bones (Tb. N) (1/mm) *p < 0.05. **(E)** The observation of tumor morphology. **(F)** Weekly measurement of the tumor volume. **(G)** Measurement of the tumor mass. **(H)** H&E staining and representative immunohistochemistry (IHC) results for Ki67 and cyclin D1, and the percentage of Ki67-positive and cyclin D1-positive cells in the control and P+ groups. *p < 0.05.

According to the procedure shown in **Figure 2A**, animal models with an *in situ* OSCC and periodontitis-associated bacterial treatment were constructed. The results of micro-CT (**Figure 2B**) showed that the alveolar bone resorption of mice in the P+ group (75.3 ± 4.0 μm) increased by about 53% compared with that of the control group (48.8 ± 1.3 μm) (**Figure 2C**). Furthermore, statistical significance was detected in the number of trabecular bone (Tb. N) (1/mm) decrease (**Figure 2D**) (p < 0.05). At the second week after tumor cells were inoculated, the mice were sacrificed, and the tumor tissues were separated and weighed. The tumor mass (1.24 ± 0.15 g) in the P+ group was about 30% higher than that in the blank control group (0.95 ± 0.19 g) (**Figures 2E, G**). At the same time, the tumor volume was measured by Vernier calipers every week, and we found that the tumor growth rate of the P+ group was higher than that of the control group (**Figure 2F**).

H&E staining of isolated tumors showed that large nuclei with different shapes, large abnormalities, and active abnormal mitotic phenomena were found in both groups (**Figure 2H**). Compared with the control group, large-scale flaky necrosis was found in the tumor tissue of the P+ group. The unclear cell outline and the fragmented and dissolved nucleus were in the necrotic area. Damaged vascular profile and a lot of cell debris were also found. In the non-necrotic area, abnormal and irregular arrangement of the cells was found, but the morphology of tumor cells was complete and clear.

**Figure 3:**
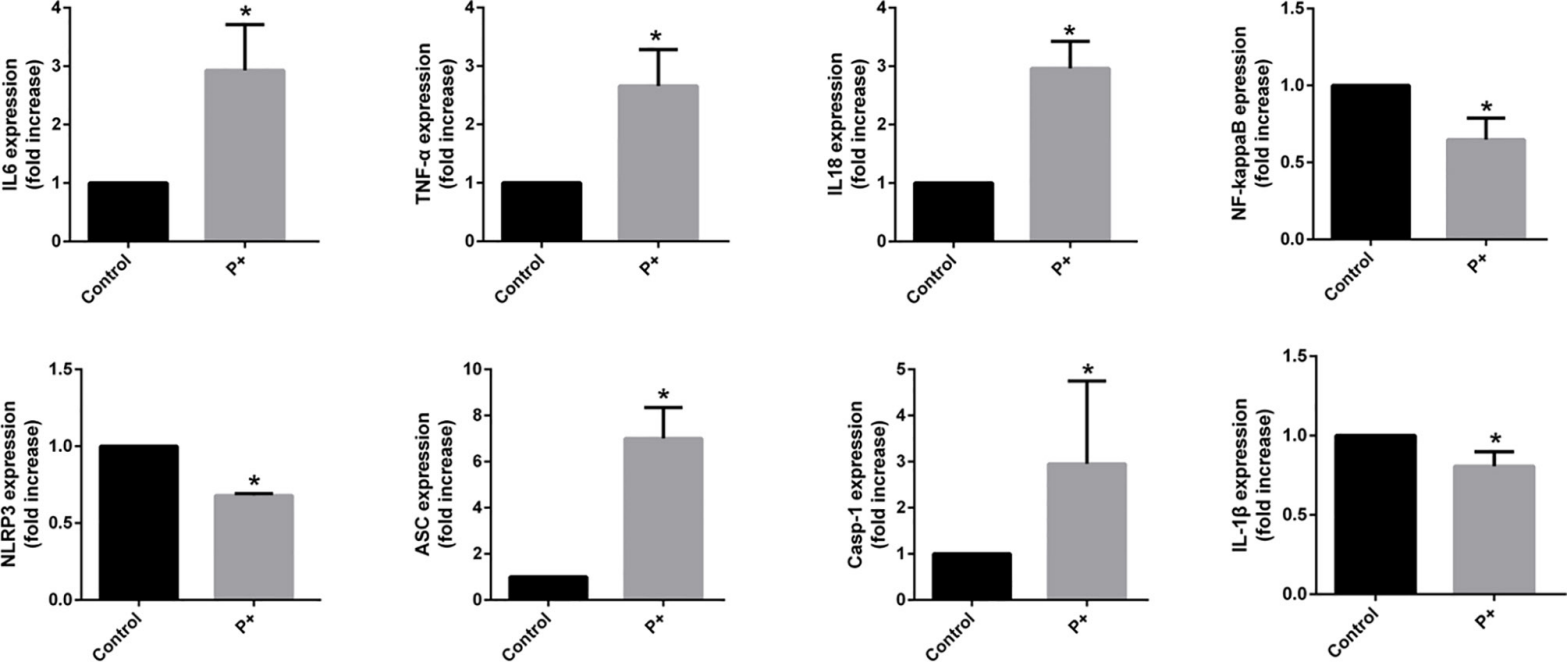
The qRT-PCR results of the expression of inflammatory cytokines in oral tumor-bearing mice, including interleukin (IL)-6, tumor necrosis factor (TNF)-α, nuclear factor (NF)-κB, NOD-, LRR- and pyrin domain-containing protein 3 (NLRP3), apoptosis-associated speck-like protein containing a CARD (ASC), caspase-1, IL-1β, and IL-18. *p < 0.05.

IHC was utilized to detect the expression of Ki67 related to tumor proliferation. A brownish black nucleus indicates positive Ki67 expression. Consistent with the phenotypic trend of the tumor tissues, the results of IHC showed that the expression of Ki67 was significantly higher in tumor tissues of the P+ group (33.19% ± 4.28%), about 1.5 times that of the control group (20.38% ± 2.54%) (p < 0.05). In addition, the positive rate of cyclin D1 was significantly higher in tumor tissues of the P+ group (30.81% ± 6.33%), approximately 2.5 times that of the control group (11.69% ± 3.58%) (p < 0.05) (**Figure 3**).

### Effect of Periodontitis-Associated Bacteria on the Expression of Inflammatory Cytokines in Oral Tumor-Bearing Mice

To evaluate the expression of inflammatory cytokines in oral cancer, total RNA was isolated from snap-frozen tumor tissues. The qRT-PCR results of the expression of inflammatory cytokines in oral cancer showed that periodontitis-associated bacteria significantly (p < 0.05) upregulated IL-6, TNF-α, IL-18, ASC (up to six times), and caspase-1 (up to four times), but it downregulated NF-κB, NLRP3, and IL-1β (less than 0.5 times).

### Histological and Immune Characteristics of the Spleen

**Figure 4:**
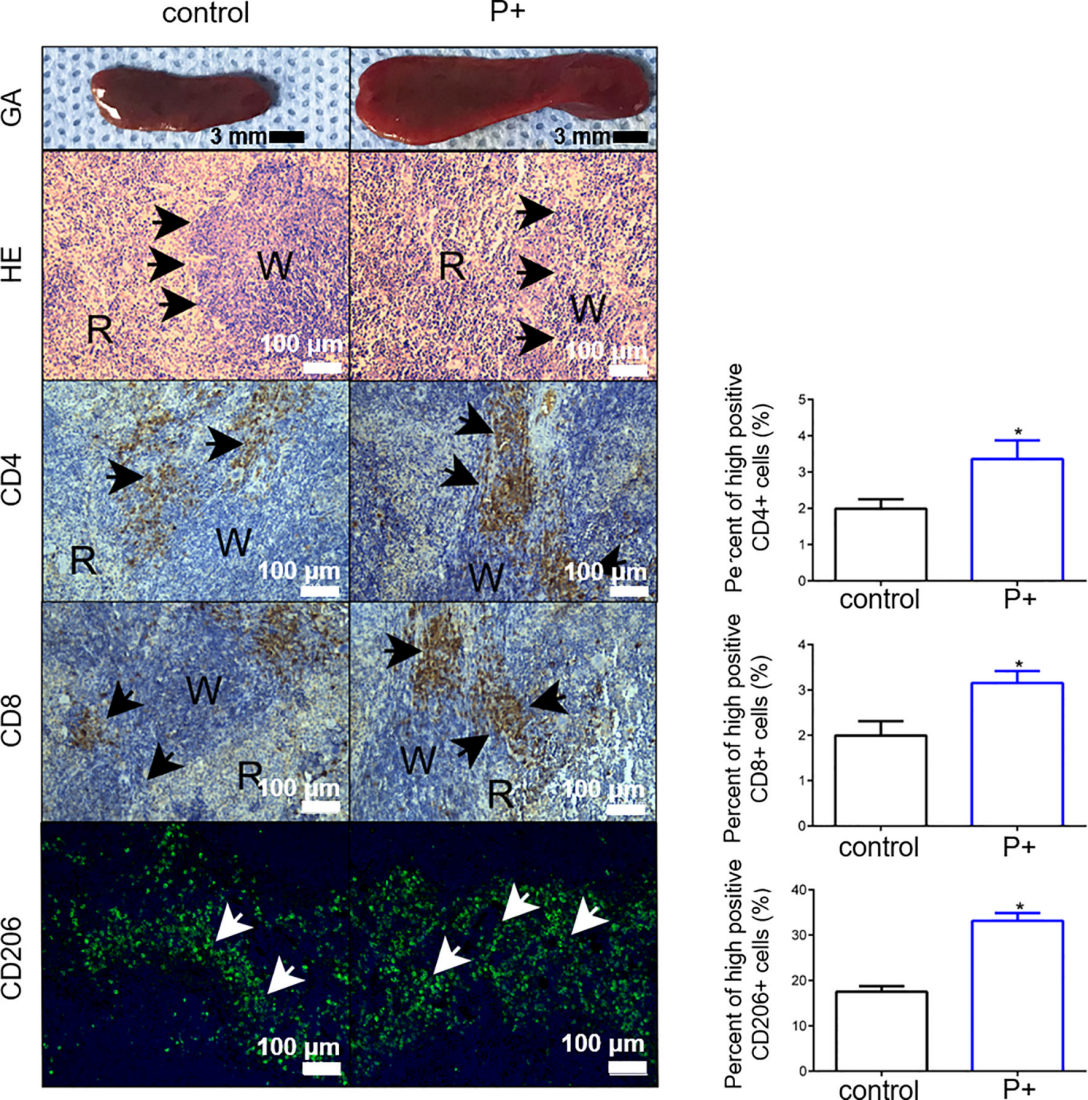
The measurement of the volume of spleen tissue and H&E staining were performed to observe histological characteristics. The changes of CD4+ T cells, CD8+ T cells, and CD206+ T cells were detected for immune characteristics of the spleen. *p < 0.05.

The volume of spleen tissue in the P+ group increased 2–3 times significantly compared with that in the control group. To determine whether periodontitis-associated bacterial infection altered the histological characteristics of the spleen, H&E staining was performed. The H&E staining results showed that compared with control group, diminution of white pulp and congestion of red pulp were found in P+ group (shown by black arrows). Moreover, we detected the change of three types of immune cells in the spleen of the tumor-bearing mice, including CD4+ T cells, CD8+ T cells, and CD206+ T macrophages (**Figure 4**). The levels of those cells were upregulated 1.5 times by periodontitis-associated bacteria (p < 0.05).

### The Periodontitis-Associated Bacteria Induce the ATR-CHK1-Dependent DNA Damage Response in Oral Tumor-Bearing Mice

**Figure 5:**
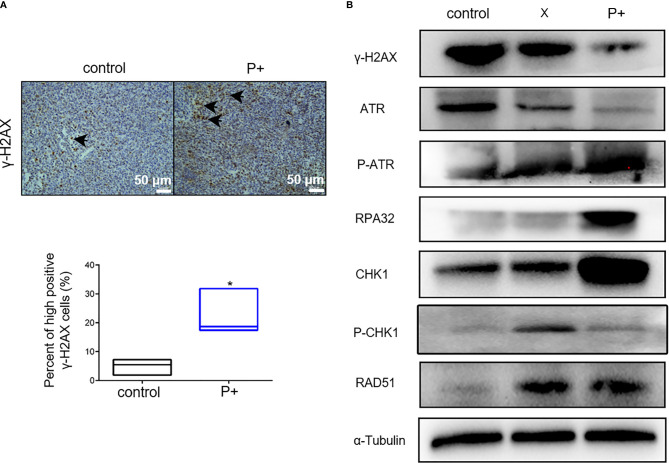
The periodontitis-associated bacteria induce the ATR-CHK1-dependent DNA damage response in oral tumor-bearing mice. **(A)** The expression of γ-H2AX in oral tumor-bearing mice was detected by immunohistochemistry. *p < 0.05. **(B)** The expression levels of γ-H2AX, ATR, p-ATR, RPA32, CHK1, p-CHK1, RAD51, and α-Tubulin in tumor tissues were performed in Western blotting.

The effect of periodontitis-associated bacteria on the expression of γ-H2AX in oral tumor-bearing mice was detected by immunohistochemistry. As shown in **Figure 5A**, the number of positive cells in the P+ group increased significantly, which was about three times that of the control group (*p < 0.05). In agreement, the results of Western blotting in tumor tissues showed that expression levels of γ-H2AX, p-ATR, RPA32, CHK1, and RAD51 were upregulated, and the phosphorylation level of CHK1 (p-chk1) was downregulated (**Figure 5B**).

## Discussion

In this study, we intended to explore the effect of periodontitis-related bacteria on the biological behavior of OSCC, including the expression of inflammasome NLRP3 and the potential mechanism of regulating ATR-CHK1-dependent DNA damage *in vivo*. It was found that *P. gingivalis* and *F. nucleatum* could promote the proliferation of OSCC and S phase cell cycle arrest, inhibit apoptosis, activate upstream signal molecules of ATR-CHK1, inhibit the activation of CHK1, and initiate the overexpressed NLRP3.

Chronic periodontitis was a risk factor for oral precancerous lesions and cancer. To date, many studies have suggested a link between periodontitis-related bacteria and oral cancer (17). Recently, microbial dysbiosis has been reported to promote oral tumorigenesis (18). Research focused on the “red complex”: *P. gingivalis*, *Treponema denticola*, and *Tannerella forsythia*, a prototype polybacterial pathogenic consortium in periodontitis. The representative bacteria of “yellow complex,” *F. nucleatum*, also received more and more attention for the close relationship with the occurrence and development of many tumors. In particular, the combined “red and yellow complex” (*P. gingivalis* and *F. nucleatum*) has been proposed as a potential etiological agent for oral cancer due to its induction of pro-inflammatory cytokines, cell proliferation, cell survival, cell invasion, and cell migration (19–21). Therefore, these authors collected comprehensive data to contribute the association between OSCC and periodontal bacteriome. It was found in this study that *P. gingivalis* and *F. nucleatum* could promote the proliferation of HSC-3 cells *in vitro* and increase the arrest of S phase cells, similar to previous studies (22, 23). The increase of S phase arrest of cell cycle increased the replication pressure of tumor cells, stimulated the probability of genome mismatch, and promoted the progress of the tumor (24). It was also found in this study that *P. gingivalis* and *F. nucleatum* can promote the expression of cyclin D1, which can promote the cell cycle G1–S transition, leading to the increase of S phase arrest. The similar results were shown in the research on gingival epithelial cells infected by *P. gingivalis* (23). In the primary culture of short-term gingival epithelial cells, *P. gingivalis* can inhibit apoptosis by upregulating antiapoptotic molecule Bcl-2 and downregulating pro-apoptotic Bad (25). The effect of *P. gingivalis* on the apoptosis of oral epithelial cells still needed further research. Some studies have found that when *P. gingivalis* invaded oral cancer cells, the autophagy increased, rather than the apoptosis (26). On the contrary, *P. gingivalis* and *F. nucleatum* can inhibit the apoptosis of HSC-3 cells and promote the survival of tumor cells in this study. In our study, the combined “red and yellow complex” (*P. gingivalis* and *F. nucleatum*) was given to the cells to elucidate the mechanism between periodontitis and oral tumors tentatively, as the solely bacterial infection has been fully researched. As a consequence, the specific mechanism might be related to the DNA damage and inflammasome NLRP3. However, the dental plaque, the etiological agent for dental caries and periodontal disease, was an archetypical biofilm composed of a complex microbial community. *P. gingivalis* and *F. nucleatum* are only a part of the complex microbial community. It was tough to elucidate the mechanism between periodontitis and oral tumors comprehensively through a single study of these two bacteria alone or in combination.

DNA damage in host cells caused by genetic toxicity of microorganisms can initiate the overexpressed ATR-CHK1 and the activated DDR to block the progress of cell cycle. Different virulence factors had different regulatory effects on DDR (27, 28). However, most of these cell cycle captures were transient. With the aggravation of DNA damage and the proliferation of cells, the gene mutations and genomic instability increased, promoting the occurrence and development of tumors (29). *In vitro*, it was found that P+ group could induce DNA damage in OSCC cells under short-term acute infection, which made γ-H2AX gradually accumulate with cell proliferation, activate the upstream signal molecule of ATR-CHK1, and increase the phosphorylation level of ATR and RPA2. RPA was recruited to single-stranded DNA fragments in response to replication stress, resulting in recruitment and activation of ATR (30). In addition to the increase in the level of autophosphorylation of ATR, the increase of RPA32 phosphorylation has been used as a reliable marker of ATR activation (31). These results were consistent with the previous studies on the regulation of DNA damage in human trophoblast cells by *P. gingivalis*. In addition, cytolethal distending toxin (CDT) had a similar effect on fibroblasts (32, 33). Usually in response to replication stress, p-CHK1 activated the cell cycle checkpoint and promoted DNA damage repair (30). In contrast, it was found in this study that the expression of CHK1 in P+ group increased, but that of p-CHK1 decreased. Due to the inhibition of CHK1 phosphorylation, the interruption of DDR signal transduction, and the inactivation of cell cycle detection points, a large number of damaged DNA substances entered mitosis and genomic instability increased, promoting the occurrence of cancer. Our results were similar to the results of a recent study that Epstein–Barr (EB) virus promoted the deletion of claspin through signal transducer and activator of transcription (STAT)3, which interrupted ATR-Chk1 signal transduction and promoted tumorigenesis (34). However, whether there were other proteins involved in the inhibition of CHK1 phosphorylation by P+ group needs to be further studied. The protein expression was mainly affected by transcriptional translation and the balance of protein stability and degradation (35). It was found that the total protein of ATR was downregulated and the total protein of CHK1 was upregulated, but upregulated at the level of mRNA, indicating that P+ group promoted the degradation of ATR protein. However, CHK1 promoted the transcriptional level to achieve the effect of upregulation. The high expression of CHK1 can also promote the arrest of S–G2M phase of cell cycle (36). The results of *in vivo* experiments were similar. The similar results were found in the colon cancer cells treated with the CDT. The CDT increased the number of cells with chromosome aberration and decreased their response to DNA damage, resulting in a decrease in their ability to produce γ-H2AX (37). In clinical studies, it was also found that the expression of γ-H2AX was upregulated in gastric precancerous lesions. However, the expression in gastric cancer tissue samples was significantly lower than that in precancerous lesions (38). As a consequence, it was speculated that the downregulation of γ-H2AX in P+ group *in vivo* may be due to the long-term chronic damage of periodontal pathogens, resulting in the downregulation of tumor cells’ response to DNA damage. In addition, the inhibition of CHK1 phosphorylation level in P+ group was more obvious, indicating that chronic stimulation could enhance the inhibitory effect.

Inflammasomes can form a polyprotein complex by combining apoptotic spot-like protein (ASC) and caspase-1 precursor (procaspase-1) with receptor NLRP3 as scaffold. It is the platform of caspase-1 activation. In addition, it can also be activated by NF-κB, which controls the maturation and secretion of IL-1β and IL-18 and initiates the inflammatory response (39). Recent research showed that NLRP3 was overexpressed in tumor tissues from head and neck squamous cell carcinoma (HNSCC), and its activity levels also correlated with tumor size, lymph node metastatic status, and IL-1β expression, which played a pro-tumorigenic role and enhanced the aggressiveness of HNSCC (40, 41). Periodontal pathogens might contribute to HNSCC pathogenesis by increasing the IL-1β response due to inflammasome dysregulation. *P. gingivalis* and *F. nucleatum* increased IL-1β by upregulating inflammasome AIM2 and downregulating POP1, and *Pg* alone promoted IL-1β by upregulating NLRP3 (20). Inflammasome NLRP3 played an important role in the inflammatory response of gingival epithelial cells (42). In addition, the expressions of NLRP3, IL-1β, and IL-18 in gingival tissues of patients with gingivitis, invasive periodontitis, and chronic periodontitis were significantly increased (43). Moreover, periodontal pathogens could regulate the expression of NLRP3 inflammasome in gingival fibroblasts (44). As an important regulator of inflammation and cell death, inflammasomes not only were related to immune inflammatory diseases but also played an important role in promoting the development of “normal tissues to chronic inflammation to malignant tumors” (45, 46). *P. gingivalis* could initiate activation of the ATP–P2X7 pathway by regulating NLRP3 that promoted the antiapoptotic enzyme NDK secretion and secreted various virulence factors, inducing the occurrence of OSCC. NLRP3 inflammasome activation had been shown to activate cancer stem cells (CSCs) leading to self-renewal and acceleration of HNSCC progression (4, 47). In addition, periodontitis-related bacteria could also promote the proliferation and invasion of tumor induced by chemical drugs in mice (48). Inflammasomes played a significant contrastive role in the interaction between malignant tumor cells and their microenvironment. In our study, the expression of inflammatory cytokines in OSCC showed that periodontitis-associated bacteria significantly (p < 0.05) upregulated IL-6, TNF-α, IL-18, ASC (up to six times), and caspase-1 (up to four times) but downregulated NF-κB, NLRP3, and IL-1β (less than 0.5 times). Considering the heterogeneity of tumor cells, the expression of NLRP3 inflammasome in the P+ tumor tissues was mainly activated. The caspase-1 activation can cause inflammatory response and carcinogenesis in inflammatory cells. As for the inconsistent expression of cytokines, we were more concerned about the heterogeneity of tumor cells, which included a variety of cells, and the cell-to-cell difference in the expression of inflammasomes or other inflammatory cytokines existed. In the later stage, the cytokine chip or single-cell sequencing of tumor tissue could be a potential solution. Furthermore, elevated NLRP3 expression is associated with chemoresistance in the treatment of HNSCC (49). The inhibition of the NLRP3 inflammasome pathway was suggested to be a promising approach for decreasing tumor cell invasion and survival.

In addition, there was a close connection between the NLRP3 inflammasome activation and reactive oxygen species (ROS) (50). On the one hand, ROS was shown to be a critical mechanism triggering NLRP3 inflammasome formation and activation in response to many exogenous stimuli as well as damage-associated molecular patterns (DAMPs). The inhibition of reduced nicotinamide adenine dinucleotide phosphate (NADPH) oxidase-derived ROS prevented ATP-induced caspase-1 activation and IL-1β production in alveolar macrophages. In particular, *P. gingivalis*-induced ROS production has been shown to activate the NLRP3 inflammasome in macrophages, leading to an increased production of atherogenic cytokines such as IL-1β, IL-18, and TNF-α (51). During the *F. nucleatum* infection, the depletion of NLRX1, a member of the NLR (NOD-like receptor) family, by shRNA attenuated ATP-induced mitochondrial ROS generation and redistribution of the NLRP3 inflammasome adaptor protein, ASC (52). On the other hand, when NLRP3 inflammasome activation was stimulated by hypercholesterolemia, DAMPs, and so on, the local inflammatory response occurred, and there were the recruitment and activation of inflammatory cells such as macrophages and T cells, producing cytokines and ROS, resulting in chronic sterile inflammation and leading to tissue injury and sclerosis (50). In our follow-up research, we would further pay attention to the potential mechanisms of the occurrence and development of oral tumor under the action of periodontal microorganisms due to ROS, inflammasome, and DNA damage.

Moreover, there were data suggesting that inflammasome polymorphisms were closely related to DDR, influencing the initiation and development of the disease’s lesions. The overexpressed NLRP3 could mimic silica-induced DNA damage and mutagenic double-strand breaks that were documented as increased levels of γ-H2AX, p-CHK2 in airway epithelial cells and closely related to the occurrence of lung carcinomas (23). Moreover, mutations in AIM2 led to excessive accumulation of DNA damage in neurons as well as an increase in the number of neurons that incorporated into the adult brain (53). Conversely, NLRP3 inflammasome activation could be initiated by DNA damage induced by ultraviolet, reactive oxygen, candidalysin, and so on (54–57). In this study, we found that overexpressed NLRP3 and DNA damage were simultaneously found in the tumor tissues of P+ group, promoting tumor growth and proliferation.

In conclusion, the periodontitis-related bacteria (*P. gingivalis* and *F. nucleatum*) can initiate the overexpressed NLRP3, activate upstream signal molecules of ATR-CHK1, and inhibit the activation of CHK1, promoting tumor growth and proliferation. It is expected to develop a new molecular mechanism between periodontitis-related bacteria and OSCC.

## Data Availability Statement

The raw data supporting the conclusions of this article will be made available by the authors, without undue reservation.

## Ethics Statement

The animal study was reviewed and approved by the ethics committee of West China Hospital of Stomatology, Sichuan University (WCHSIRB-D-2020-394).

## Author Contributions

YY, XS and BT conceived and designed the research studies, analyzed the data, and wrote the manuscript. YY, XS, and MZ performed the experiments. All authors contributed to the article and approved the submitted version.

## Funding

This study was supported by the Innovation and Entrepreneurship Training Program for College Students (grant no. C2019105015) and Open Project Fund for Key Laboratory of Guangdong Science and Technology Department (KF2019120101).

## Conflict of Interest

The authors declare that the research was conducted in the absence of any commercial or financial relationships that could be construed as a potential conflict of interest.

## Publisher’s Note

All claims expressed in this article are solely those of the authors and do not necessarily represent those of their affiliated organizations, or those of the publisher, the editors and the reviewers. Any product that may be evaluated in this article, or claim that may be made by its manufacturer, is not guaranteed or endorsed by the publisher.
