# Supplementary material for: Periodontal Pathogens Promote Oral Squamous Cell Carcinoma by Regulating ATR and NLRP3 Inflammasome
Source: Front Oncol. 2021 Sep 30;11:722797. doi: 10.3389/fonc.2021.722797 (PMC8514820; doi:10.3389/fonc.2021.722797)
Supplement: Supplementary file 1 [file Table_1.doc]

**Supplementary Table 1 Primer sequence**

| **Gene Name** | **Gene ID** | **Size (bp)** | **Sequence (5’ to 3’)** |
| --- | --- | --- | --- |
| IL-6  IL-18 | 16193  16173 | 6862  27142 | F: CTCCCAACAGACCTGTCTATAC  R: CCATTGCACAACTCTTTTCTCA  F: AGACCTGGAATCAGACAACTTT |
|  |  |  | R: TCAGTCATATCCTCGAACACAG |
| NF-κΒ | 4790 | 116037 | F: CAAAGACAAAGAGGAAGTGCAA |
|  |  |  | R: GATGGAATGTAATCCCACCGTA |
| TNF-α | 100136034 | 2002 | F: TCTTCTGCCTGCTGCACTTTG |
|  |  |  | R: CGAGATAGTCGGGCCGATTG |
| NLPR3 | 216799 | 25387 | F: ATGTGAGAAGCAGGTTCTACTC |
|  |  |  | R: CTCCAGCTTAAGGGAACTCATG |
| ASC | 110333045 | 1301 | F: AGAGGATTTCTTAACGGATGCA |
|  |  |  | R: TCACAAGACCAGGCATATTCTT |
| caspase-1 | 12362 | 8765 | F: ACAATGACTGTGCTTAGAGACA |
|  |  |  | R: CACAGCTCCAGACTCTTCTTTA |
| IL-1β | 16176 | 6570 | F: CAACTGTTCCTGAACTCAACTG |
|  |  |  | R: GAAGGAAAAGAAGGTGCTCATG |
| GAPDH | 14433 | 4616 | F: AAGGTCGGAGTCAACGGATT |
|  |  |  | R: CTCCTGGAAGATGGTGATGG |
